# Understanding childbirth as a complex salutogenic phenomenon: The EU COST BIRTH Action Special Collection

**DOI:** 10.1371/journal.pone.0236722

**Published:** 2020-08-05

**Authors:** Soo Downe, Jean Calleja Agius, Marie-Clare Balaam, Lucy Frith

**Affiliations:** 1 ReaCH Group, UCLan, Lancashire, United Kingdom; 2 Department of Anatomy, Faculty of Medicine and Surgery, University of Malta, Msida, Malta; 3 Institute of Population Health, University of Liverpool, Liverpool, United Kingdom; PLOS, UNITED KINGDOM

## Abstract

In 2014, the EU funded a four-year European Cooperation in Science and Technology (COST) Action to address the topic of childbirth. The COST Birth Action was a cross-European network, that brought together over 120 scientists, practitioners, activists and policy makers from 34 countries to work on intrapartum care. The central aim was to advance the state of research and practice in a specific area of great clinical and social importance, intrapartum care. The Action used inter and trans-disciplinary approaches to address birth from multiple perspectives and drew on complexity theory and the concept of salutogenesis (wellbeing). This special collection presents six papers produced from the Action and gives a sense of the range and depth of the work conducted. The Collection illustrates the knowledge that can be generated when a diverse group of people come together with a similar goals and perspectives.

## Underpinning theory of the action

The underpinning scientific framework of the Action [1] was complexity theory, applied through the lens of Sackett’s multi-system complex adaptive notion of EBM. In contrast to most research in the intrapartum period, it was also designed to explore childbirth from a salutogenic (wellbeing) perspective, with a particular focus on the salutogenic concept of ‘sense of coherence’.

### Complexity theory

The promise of classical enlightenment science has been that scientific method based on the idea that A causes B, (a linear hypothesis) can be used to uncover what is true. The belief underpinning this hypothesis is through scientific progress, we will eventually know everything there is to know about the world [2]. The academic and engineering discoveries resulting from this approach led to the marvels of the industrial revolution and a huge number of social and health care improvements. However, over the last thirty years or so there has been a significant move away from reliance on simple linear calculations undertaken in disciplinary silos, and towards a new kind of inter or even trans-disciplinary science based on complex adaptive theory [3].

New ideas, insights, and phenomenon that emerge unexpectedly are fundamental to this new approach. The parts of all kinds of different systems (the weather, flowers, the sea, neurobiology and so on) interact, and organise themselves spontaneously into new, unpredictable patterns and characteristics. The consequence is that the overall system cannot be predicted or described based only its component parts. Emergence arises when self-organising components of a complex adaptive system interact. These generate new unpredictable pathways as a consequence of this interactivity [4]. Often these patterns are fractal, that is, similar patterns tend to emerge at different levels of the system [5]. Work on complex adaptive system analysis from a range of different disciplines has culminated in new insights into emergent non-linear behaviours of systems as diverse as evolution [6], organisations [7] and climate [8]. Interest in complexity theory is also increasing amongst health researchers, especially in implementation science [9].

Evidence-based medicine (EBM) has become central to modern healthcare policy and practice. This is often interpreted through protocols and guidelines derived from systematic reviews of population level data, and then applied to all those with particular conditions/clinical needs [10]. David Sackett, one of the key architects of EBM, framed EBM in a different way. He and his colleagues saw EBM as an integration of multiple systems of knowledge, values and expertise that, together, produce a unique optimal outcome for each clinical encounter:

*Evidence-based medicine (EBM) is the integration of best research evidence with clinical expertise and patient value*… *when these three elements are integrated*, *clinicians and patients form (an)…alliance which optimises clinical outcomes and quality of life…’*

[11]

### Salutogenesis

The theory of salutogenesis was developed by Aaron Antonovsky [12]. It conceptualises peoples’ capacity for wellbeing in terms of how they experience events. The degree to which people see the world as meaningful, manageable and comprehensible is characterised by their Sense of Coherence (SoC). High SoC tends to be associated with better health and social outcomes. Antonovsky’s theory relates to notions of human flourishing and a turn towards this kind of thinking can be seen in the United Nations’ shift from a focus on survival alone towards thriving and transformation for women, girls, and adolescents [13]. In this context, childbirth becomes more than just a means of getting the baby out of the mother’s body safely. It becomes a potential space for positive transformation for mother and baby neurohormonally, psychologically, physically, and emotionally throughout the life-course.

## Current norms in childbirth and related research

There are hundreds of thousands of published studies in the area of pregnancy, labour and birth. The vast majority of these are focused on pathologies and the interventions designed to prevent them. This would be an uninteresting observation, if the field of investigation was a particular disease, illness, or adverse state. Indeed, for women and babies who do have diseases or complications, this type of research is critical. For example, the evidence on the efficacy of magnesium sulphate to prevent pre-eclampsia and to treat eclampsia [14] and the use of tranexamic acid for catastrophic haemorrhage [15] have saved many lives around the world. However, the large majority of the 130 million women who give birth annually do not require medical procedures or pharmacological agents to safely complete a process that has been shaped by complex adaptive evolution—a process that is critical to the survival of the human race.

In 1978, the Alma Alta agreement stated:

*Health is*…*a state of complete physical*, *mental and social well-being and not merely the absence of disease or infirmity"*

[16]

In terms of childbirth, this has been reinforced by the following statements from an obstetrician (Montgomery 1958) and from the UK NICE guidelines (2020). The NICE guidelines were written 60 years after Montgomery made the following comment:

*I have stated on numerous occasions that there is no more need to interfere with the course of normally progressing labor than there is to tamper with good digestion*, *normal respiration*, *and adequate circulation*

[17]

*Do not offer or advise clinical intervention if labour is progressing normally and the woman and baby are well*. *In all stages of labour*, *women who have left the normal care pathway because of the development of complications can return to it if/when the complication is resolved*.

*(NICE)* [18]

Despite this, a search of PubMed undertaken on February 15^th^ 2020 using the term ‘childbirth physiology’ to capture all articles on the physiology of childbirth only generated 17,131 hits. In contrast, searching for studies of a specific procedure ‘caesarean section’, generated 62,945 hits. This suggests that there is four times as much research taking place on one single surgical childbirth intervention than for the whole field of straightforward, uncomplicated labour and birth.

Rates of routine intervention in childbirth, such as induction and augmentation of labour, surgical birth, and widespread routine use of antibiotics for mother and baby [19], are high and rising around the world. In parallel, inequalities in access to such treatments for marginalised, poor, rural, and/or ethnic minority groups are evident globally, within and between countries [20]. Most global experts and agencies agree that there is an imbalance in maternity care around the world. This can be characterised as *‘too little too late’* for many marginalised, poor and/or rural women and babies, and *‘too much too soon’* for many urban women and neonates. In even the poorest economies, the inverse care law operates. More marginalised women who need lifesaving interventions for them or for their babies are not getting them. Whereas, wealthier women who do not need such interventions are getting too many of them. This can lead to iatrogenic harm for them and their babies in the short and longer term [21]. For example, the USA, has a very high rate of spending on interventions in normal pregnancy and birth [22], and caesarean sections are very common [23]. However, their national maternal mortality rate, 17.4/100,000, is the highest among all high-income countries. This is a higher rate than many middle-income countries [24]. There is also intra-country variation in maternal mortality rates; in the state of Louisiana the rate has been cited at around 12/100,000, in contrast to California, where the rate is 4/100,000 [25]. In Europe, according to Euro-Peristat, caesarean section rates in 2015 ranged from 16.1% (Iceland) to 56.9% (Cyprus), with no evidence that the highest rates are correlated with improved outcomes for mother and baby when compared to countries with the lowest rates [26]. Preterm birth and postpartum haemorrhage rates are also rising in Europe and in other high-income countries [27, 28].

These data suggest that, while the current way of doing birth around the world has brought benefits to some women and babies, it is also associated with harms for others. This is particularly prevalent in privately funded health care systems, or in countries where access to good health care, education, housing and women’s rights are restricted [29]. The drivers that generate this situation are complex, intersectional, and multifactorial. Under these conditions, classical ways of framing and undertaking research in maternity care, that assume that standardised drugs, treatments, or protocols that work at the population level will work for every individual in every context, do not seem to be optimally effective.

An illustration of this is the assumption that universal hospitalisation for giving birth will inevitably reduce maternal and infant mortality and morbidity, and will improve long term physical and psychological thriving. This assumption has been only partially evident in practice. Indeed, there is evidence from a number of high-income countries that hospitalisation of healthy women and babies without complications can increase iatrogenic damage for women and does not benefit their babies. The increased use of induction, augmentation, and caesarean section contribute to these iatrogenic harms [30–32]. Some (but not all) studies in this field have demonstrated longer-term epigenetic and microbial impacts associated with these interventions. These have been linked to rises in auto-immune diseases, such as type one diabetes, and some childhood leukaemia types [33–35].

Balancing the benefits of non-intervention for physiologically normal childbirth with the need to intervene appropriately when pathology arises in the complex, dynamic, highly individual situation of labour could be termed a ‘wicked’ problem. Such problems are defined as *dilemmas in political and social planning that resist clear definitions*, *defy traditional analysis approaches*, *and refuse definitive resolution through predetermined solutions* [36].

Intriguingly, as an exemplar of the way in which wicked problems do not have simple right or wrong solutions, a recent study of 64 facilities in Ghana showed a complex set of relationships between maternal and neonatal mortality and factors such as geographic distance from facilities, wealth, and education. These factors influence maternal and neonatal mortality in unexpected directions [37]. Wealthier women were more likely to attend hospital for birth, but their outcomes were not better for them or their babies when compared to poorer women who did not attend facilities. In fact, the best outcomes were obtained for women who attended facilities that provided BOTH effective emergency care for the minority who needed it, AND the capacity to protect physiological labour and birth for the majority of women and babies for whom this was appropriate. Further, at the basic science level, a recent study of the postnatal microbiome of babies compared babies born in hospital and at home, who had completely straightforward births without procedures, pharmacological agents, or water birth. The researchers found clear differences in the gut colonisation of the neonates born at home when compared to those born in hospital, and concluded:

*Hospitalization (perinatal interventions or the hospital environment) may affect the microbiota of the vagina… and… initial colonization during labor and birth*, *with effects that could persist in the intestinal microbiota of infants 1 month after birth*.

[38]

This suggests that physiological birth in a home/community environment is a critical contextual mediator of the biological adaptive processes of labour and birth. The consequences of this, both in the short and long-term are not yet fully understood.

## Implications for the COST Birth Action

In recognition of the potential intrinsic value of physiological labour and birth as a catalyst for long-term wellbeing, those collaborating on the COST Birth Action set out to re-imagine the kinds of questions and investigations that could be asked and undertaken in the future. The mix of academic and clinical disciplines, policy makers, service users, and activists who were invited to join the Action at the outset, and who joined it subsequently, worked to unsettle current scientific and normative ways of understanding and investigating this topic area. The salutogenic approach taken by the Action entailed examining how things go right, as well as how they go wrong, with an aim of increasing research on physiology as well as pathology.

The papers in this Special Collection are indicative not only of the inter and trans-disciplinary insights that emerged from the interaction and multiple perspectives of over 120 people from 34 countries over the four years of working on the EU COST Action. They also illustrate the new kinds of questions that can be asked once those from different disciplines and perspectives spend time listening to each other. The Action worked across disciplines, challenging assumptions and incorporating service users and activists’ experiences and knowledges. The papers in this Collection on neurophysiology (both the psychology of neurophysiological events, and oxytocin levels related to breastfeeding and birth interventions) address some of the basic science questions that still remain to be explored in the delicate neurohormonal-psycho-social dance that labour has evolved to be [39, 40]. The investigation of thermal imaging as a basis for understanding how physiological labour works is an illustration of the synthesis of knowledge across clinical practice, imaging science, and service user engagement [41, 42]. Understanding how midwives work to facilitate physiological birth provides a window on techniques and practices that have, heretofore, been somewhat hidden [43]. The examination of different oxytocin regimes across Europe challenges assumptions that induction and augmentation guidelines are based on sound knowledge of physiological labour processes [44]. Finally, the economic analysis of variations in caesarean rates illustrates how context influences data and their analysis and interpretation [45].

## Conclusion

Members of the EU COST Birth Action engaged with childbirth from the perspectives of both complexity theory and salutogenesis. The networks formed by the scientists, academics, clinicians, service users, activists and policy makers that were included in the programme have generated unexpected, emergent insights, that have led to new areas of enquiry that are ongoing. The intention of this Collection is to summarise and synthesis some of the research in this area and contribute to growing the evidence base in this critical, but relatively neglected, field of physiological labour and birth.
